# Chromosome-level genome assembly of Norwegian wild alpine reindeer (*Rangifer tarandus tarandus*)

**DOI:** 10.1093/jhered/esaf094

**Published:** 2025-11-03

**Authors:** Ole K Tørresen, Ave Tooming-Klunderud, Morten Skage, Anne Eline Streitlien, Olav Strand, Christer M Rolandsen, Giada Ferrari, José Cerca, Atle Mysterud, Kjetill S Jakobsen

**Affiliations:** Centre for Ecological and Evolutionary Synthesis (CEES), Department of Biosciences, University of Oslo, P.O. Box 1066 Blindern, NO-0316 Oslo, Norway; Centre for Ecological and Evolutionary Synthesis (CEES), Department of Biosciences, University of Oslo, P.O. Box 1066 Blindern, NO-0316 Oslo, Norway; Centre for Ecological and Evolutionary Synthesis (CEES), Department of Biosciences, University of Oslo, P.O. Box 1066 Blindern, NO-0316 Oslo, Norway; Streitlievegen 131, 2580 Folldal, Norway; Norwegian Institute for Nature Research (NINA), P.O. Box 5685 Torgarden, NO-7485 Trondheim, Norway; Norwegian Institute for Nature Research (NINA), P.O. Box 5685 Torgarden, NO-7485 Trondheim, Norway; Centre for Ecological and Evolutionary Synthesis (CEES), Department of Biosciences, University of Oslo, P.O. Box 1066 Blindern, NO-0316 Oslo, Norway; Centre for Ecological and Evolutionary Synthesis (CEES), Department of Biosciences, University of Oslo, P.O. Box 1066 Blindern, NO-0316 Oslo, Norway; Department of Bioinformatics and Genetics, Swedish Museum of Natural History, Box 50007, SE-104 05 Stockholm, Sweden; SciLifeLab, Karolinska Institutet Science Park, Box 1031, 17121 Solna, Sweden; Centre for Ecological and Evolutionary Synthesis (CEES), Department of Biosciences, University of Oslo, P.O. Box 1066 Blindern, NO-0316 Oslo, Norway; Norwegian Institute for Nature Research (NINA), P.O. Box 5685 Torgarden, NO-7485 Trondheim, Norway; Centre for Ecological and Evolutionary Synthesis (CEES), Department of Biosciences, University of Oslo, P.O. Box 1066 Blindern, NO-0316 Oslo, Norway

**Keywords:** Earth Biogenome Project Norway, haplotype-resolved, HiFi sequencing, Rondane population, wild reindeer, Hi-C

## Abstract

We describe a chromosome-level genome assembly from a wild alpine reindeer individual (*Rangifer tarandus tarandus*) from the Rondane area in Southern Norway*.* The assembly is resolved into two pseudo-haplotypes: hap 1 spanning 3,081 Mb and hap 2 spanning 2,633 Mb. Contig N50 and scaffold N50 lengths are in the range of 31 to 41 and 66 to 69 Mb, respectively. A large part of these two haplotypes (83.8% and 90.4%, respectively) are scaffolded into 34 autosomal chromosomal pseudomolecules, and in sex chromosomes X and Y for hap 1. The BUSCO completeness scores are 98.0% and 95.2%, respectively, and gene annotations of the assemblies identified 37,998 and 36,977 protein-coding genes. We also present an updated and improved genome assembly for Svalbard reindeer (*Rangifer tarandus platyrhynchus*; contig N50 46 to 48 Mb, scaffold N50 67 to 71 Mb, BUSCO 95.4% to 98.0%) and a comparison with previously published genome assemblies of reindeer.

## 1. Introduction

Reindeer is an iconic species of the northern hemisphere playing a key role in the ecosystems and also as part of human culture (Mustonen 2022). The taxonomic delimitation of the species is somehow disputed with reindeer in Eurasia (hereafter reindeer) and caribou in North America being the same species, *Rangifer tarandus*, but typically separated into a number of subspecies (Banfield 1961; Tryland and Kutz 2018). Reindeer are distributed across the northern Holarctic region, inhabiting both arctic tundra and boreal forests. Wild reindeer populations count just below 3 million adult individuals, but numbers are declining globally and they are now Red Listed as “Vulnerable” (Gunn 2016). Reindeer conservation is important in itself, and protection of reindeer habitat can also aid in conservation of a wider range of biodiversity (Labadie et al. 2024).

Reindeer has a polygynous mating system and are sexually dimorphic in body size with males being considerably larger than females. Reindeer is the only cervid where females also possess antlers. Reindeer have a series of unique biological features enabling survival in harsh arctic environments. Reindeer are adapted to winter darkness and can see wavelengths down into the ultraviolet range (Stokkan et al. 2013), and they have specific mutations in genes involved in the vitamin D and fat metabolism pathways (Lin et al. 2019). They have a unique ability to digest and rely on a winter diet consisting of a high proportion of lichens (Falldorf et al. 2014). Reindeer have the most extreme snow coping abilities in terms of morphology among cervids (Telfer and Kelsall 1984).

Reindeer have been semi-domesticated at least two times (Røed et al. 2008) and there is evidence for milder artificial selection relative to fully domesticated species (Wu et al. 2024). Reindeer herding has been a cornerstone to the culture of several Eurasian nomadic indigenous people (Salmi 2022). Semi-domestic reindeer have also been introduced to areas in Norway, Iceland, Greenland, Alaska, and South Georgia island to live as wild, i.e. becoming feral reindeer (Mysterud et al. 2024). In Fennoscandia, semi-domestic reindeer have replaced wild reindeer from most parts of their former range, and admixture with the semi-domestics threaten the genetic integrity of the remaining wild reindeer (Anderson et al. 2017). A key issue for reindeer conservation in Europe is to save the last wild reindeer, including mainly alpine-living reindeer (*R. tarandus tarandus*) in Norway and forest-living reindeer (*Rangifer tarandus fennicus*) in Finland (Weldenegodguad et al. 2024). This is challenging due to partial mixing with semi-domestic and feral populations. Norway harbors more than ~270,000 semi-domestic and feral populations with high levels of genetic mixing with semi-domestics, but only some 8,000 wild reindeer with a low level of genetic mixing (Kvie et al. 2019).

Climate change, fragmentation, and habitat loss are well-known challenges for the conservation of reindeer (Dorber et al. 2023; Morineau et al. 2023). There are several gaps related to understanding the role of genetics critical to target conservation efforts. Fragmentation leads to smaller populations and with less gene flow to adjacent populations, and these are likely subject to increasing risk of inbreeding, drift, and loss of genetic diversity (Kvie et al. 2019; Hansen et al. 2024), similar processes as in the overharvesting of the Svalbard reindeer in 17th to 20th centuries (Kellner et al. 2024). The issue of reindeer genetics is currently also made topical due to an outbreak of chronic wasting disease (CWD) in Norway. CWD management with intensified harvesting involves severe reduction of population size, skewed age structure, and sex ratio (Mysterud et al. 2025), which may largely impact genetic processes (Kvalnes et al. 2024). A full genome assembly provides a required basis for detailed studies into selection and other processes affecting genetics of small populations.

There are several genome assemblies for reindeer ( Li et al. 2017; Dussex et al. 2023; Poisson et al. 2023), and genome assemblies at chromosome-level already exist (Poisson et al. 2023; Dussex et al. 2023). One reference genome stems from a 1-year-old male reindeer from Sodankylä, Finland (Weldenegodguad et al. 2020; Pokharel et al. 2023), representing a semi-domestic reindeer. A high-quality reference genome is available for the Svalbard reindeer (*Rangifer tarandus platyrhynchus*) (Dussex et al. 2023). This subspecies is atypical having lived on an arctic island without predators for about 5,000 years, and they are much less social, sedentary, smaller in body size, have shorter legs, store more fat, and have limited genetic variation.

Almost all of the remaining wild alpine reindeer in Europe are in Norway (Gunn 2016). Of the 24 populations managed as wild reindeer in Norway, only four of these populations (Snøhetta, Knutshø, Rondane, and Sølnkletten) have limited admixture with semi-domestics and are considered vulnerable due to ongoing habitat fragmentation and a decline in performance indicators. Here, we have generated a chromosome-level, haplotype-resolved genome assembly from an individual of the wild alpine reindeer (*R. tarandus tarandus*) from Rondane, created using PacBio HiFi long-read and Hi-C sequencing data and compare it with other publicly available genomes. This haplotype-resolved genome assembly is generated as part of the Earth Biogenome Project Norway.

## 2. Methods

### 2.1. Sample acquisition and DNA extraction

The sample was from a 3.5-year-old male reindeer from Jervetunga, Rondane, Norway (UTM32 533028, 687324). The sample was from a reindeer legally shot during the ordinary hunting season on 29 August 2023, and sampling from such a specimen requires no specific legal permit in Norway. The reference code for the sample is 100000423715 in the registry for deer data in Norway (“Hjorteviltregisteret”).

DNA isolation for PacBio long-read sequencing was performed using a QIAGEN Genomic-tip 100/G gravity-flow column with 78 mg of flash-frozen heart tissue as starting material and after the manufacturer’s instructions for tissue samples. Quality check of amount, purity, and integrity of isolated DNA was performed using the Qubit BR DNA quantification assay kit (Thermo Fisher), Nanodrop (Thermo Fisher), and Fragment Analyser (DNA HS 50 kb large fragment kit, Agilent Tech.).

### 2.2. Library preparation and sequencing for de novo assembly

Approximately 8 μg of purified HMW DNA was pre-conditioned with the DNAFluid+ kit and sheared into an average fragment size of 30 kb using the Megaruptor3 (Diagenode). For library preparation, 5 μg of fragmented DNA was used after the PacBio protocol for HiFi library preparation using the SMRTbell prep kit 3.0. The final HiFi library was size-selected with a 10 kb cut-off using a gel cassette on the BluePippin instrument (Sage Science), followed by an additional bead clean-up (PacBio) and library quantification with Qubit HS DNA quantification assay kit (Thermo Fisher). Final library size estimation was done with the Fragment Analyser kit mentioned above. Sequencing was performed by the Norwegian Sequencing Centre on the PacBio Revio instrument using the Revio polymerase kit and one 25 M SMRT cell.

A Hi-C library was prepared using the Arima High Coverage HiC kit (Arima Genomics, Inc), after the standard input protocol in the User Guide for Animal Tissues (document part number A160162 v01) and starting from 127 mg of fresh frozen heart tissue. Final library quality was assayed as above in addition to qPCR using the Kapa Library quantification kit for Illumina (Roche Inc.). The indexed library was sequenced with other libraries on the Illumina NovaSeq SP flow cell with 2 × 150 bp paired end mode at the Norwegian Sequencing Centre.

### 2.3. Genome assembly and curation, annotation, and evaluation

A full list of relevant software tools and versions is presented in Table 1. We assembled the species using a pre-release of the EBP-Nor genome assembly pipeline (https://github.com/ebp-nor/GenomeAssembly). K-mer Counter (KMC; Kokot et al. 2017) was used to count k-mers of size 32 in the PacBio HiFi reads, excluding k-mers occurring more than 10,000 times. GenomeScope (Ranallo-Benavidez et al. 2020) was run on the k-mer histogram output from KMC to estimate genome size, heterozygosity, and repetitiveness but ploidy level was investigated using Smudgeplot (Ranallo-Benavidez et al. 2020). HiFiAdapterFilt (Sim et al. 2022) was applied on the HiFi reads to remove possible remnant PacBio adapter sequences. The filtered HiFi reads were assembled using hifiasm (Cheng et al. 2021) with Hi-C integration resulting in a pair of haplotype-resolved assemblies, pseudo-haplotype one (hap1) and pseudo-haplotype two (hap2). Unique k-mers in each assembly/pseudo-haplotype were identified using meryl (Rhie et al. 2020) and used to create two sets of Hi-C reads, one without any k-mers occurring uniquely in hap1 and the other without k-mers occurring uniquely in hap2. K-mer filtered Hi-C reads were aligned to each scaffolded assembly using BWA-MEM (Li 2013) with -5SPM options. The alignments were sorted based on name using samtools (Li et al. 2009) before applying samtools fixmate to remove unmapped reads and secondary alignments and to add mate score, and samtools markdup to remove duplicates. The resulting BAM files were used to scaffold the two assemblies using YaHS (Zhou et al. 2023) with default options. FCS-GX (Astashyn et al. 2024) was used to search for putative contamination. Contaminated sequences were removed. The mitochondrion was searched for in reads using Oatk (Zhou et al. 2024). Merqury (Rhie et al. 2020) was used to assess the completeness and quality of the genome assemblies by comparing to the k-mer content of the Hi-C reads. BUSCO (Manni et al. 2021) was used to assess the completeness of the genome assemblies by comparing against the expected gene content in the mammalia and cetartiodactyla lineages. Gfastats (Formenti et al. 2022) was used to output different assembly statistics of the assemblies. The assemblies were manually curated using PretextView and Rapid curation 2.0. Chromosomes (including sex chromosomes) were identified by mapping to GCA_949782905.1 (Svalbard reindeer) in addition to inspecting the Hi-C contact map in PretextView. BlobToolKit and BlobTools2 (Laetsch and Blaxter 2017), in addition to blobtk were used to visualize assembly statistics and GC-coverage plots. To generate the Hi-C contact map image, the Hi-C reads were mapped to the assemblies using BWA-MEM (Li 2013) using the same approach as above, before PretextMap was used to create a contact map which was visualized using PretextSnapshot.

**Table 1:** Software tools: versions and sources.

Software tool	Version	Source
BlobToolKit	4.1.7	https://github.com/blobtoolkit/blobtoolkit
blobtk	0.5.8	https://github.com/blobtoolkit/blobtk
BUSCO	5.7.1	https://gitlab.com/ezlab/busco
hifiasm	0.19.8	https://github.com/chhylp123/hifiasm
KMC	3.1.2	https://github.com/refresh-bio/KMC
GenomeScope	2.0	https://github.com/tbenavi1/genomescope2.0
HiFiAdapterFilt	2.0.0	https://github.com/sheinasim/HiFiAdapterFilt
PretextView	0.2.5	https://github.com/wtsi-hpag/PretextView
PretextMap	0.1.9	https://github.com/wtsi-hpag/PretextMap
PretextSnapshot		https://github.com/wtsi-hpag/PretextSnapshot
meryl	1.3.0	https://github.com/marbl/meryl
BWA-MEM	0.7.17	https://github.com/lh3/bwa
samtools	1.17	https://github.com/samtools/samtools
YaHS	1.2a.2	https://github.com/c-zhou/yahs
FCS-GX	0.4.0	https://github.com/ncbi/fcs
Merqury	1.3	https://github.com/marbl/merqury
AGAT	1.0	https://github.com/NBISweden/AGAT
MitoHiFi	2.2	https://github.com/marcelauliano/MitoHiFi
miniprot	0.13	https://github.com/lh3/miniprot
GALBA	1.0.6	https://github.com/Gaius-Augustus/GALBA
RED	2018.09.10	http://toolsmith.ens.utulsa.edu/
Funannotate	1.8.17	https://github.com/nextgenusfs/funannotate
EvidenceModeler	2.1.0	https://github.com/EVidenceModeler/EVidenceModeler
DIAMOND	2.1.8	https://github.com/bbuchfink/diamond
InterProScan	5.62-94	https://www.ebi.ac.uk/interpro/search/sequence/
EMBLmyGFF3	2.2	https://github.com/NBISweden/EMBLmyGFF3
Rapid curation 2.0	964d17e997e00c69f25940cf96d3658bda631147	https://github.com/Nadolina/Rapid-curation-2.0

We annotated the genome assemblies using a pre-release version of the EBP-Nor genome annotation pipeline (https://github.com/ebp-nor/GenomeAnnotation). First, AGAT (https://zenodo.org/record/7255559) agat_sp_keep_longest_isoform.pl, and agat_sp_extract_sequences.pl were used on the GRCh38 genome assembly and annotation to generate one protein (the longest isoform) per gene. Miniprot (Li 2023) was used to align the proteins to the curated assemblies. UniProtKB/Swiss-Prot (UniProt Consortium 2023) release 2024_04 in addition to the vertebrata part of OrthoDB v11 (Kuznetsov et al. 2023) were also aligned separately to the assemblies. Red (Girgis 2015) was run via redmask (https://github.com/nextgenusfs/redmask) on the assemblies to mask repetitive areas. GALBA (Stanke et al. 2006; Buchfink et al 2015; Hoff and Stanke 2019; Li 2023; Brůna et al. 2023) was run with the GRCh38 proteins using the miniprot mode on the masked assemblies. The funannotate-runEVM.py script from Funannotate was used to run EvidenceModeler (Haas et al. 2008) on the alignments of GRCh38 proteins, UniProtKB/Swiss-Prot proteins, vertebrata proteins, and the predicted genes from GALBA. The resulting predicted proteins were compared with the protein repeats that Funannotate distributes using DIAMOND blastp and the predicted genes were filtered based on this comparison using AGAT. The filtered proteins were compared with the UniProtKB/Swiss-Prot release 2024_04 using DIAMOND (Buchfink et al 2015) blastp to find gene names and InterProScan was used to discover functional domains. AGATs agat_sp_manage_functional_annotation.pl was used to attach the gene names and functional annotations to the predicted genes. EMBLmyGFF3 (Norling et al 2018) was used to combine the fasta files and GFF3 files into an EMBL format for submission to ENA.

To characterize the differences between the two pseudo-haplotypes, we ran minimap2 (Li 2017) on the two pseudo-haplotypes. The resulting alignment was processed with paftools.js (packaged with minimap2), producing a report listing the number of insertions, single nucleotide polymorphisms (SNPs), and indels between the two pseudo-haplotypes.

To compare the assemblies generated in this study to other available reindeer genome assemblies, we downloaded GCA_963422255.1, GCA_902712895.1, GCA_022457185.1, GCA_019903745.2, GCA_014898785.1, and GCA_004026565.1 from NCBI, generated assembly metrics with gfastats and completeness with BUSCO and the cetartiodactyla lineage.

The text in Methods and parts of Results is based on a template we use for all the species we publish in the EBP-Nor project.

## 3. Results

### 3.1. De novo genome assembly and annotation

**Fig. 1:**
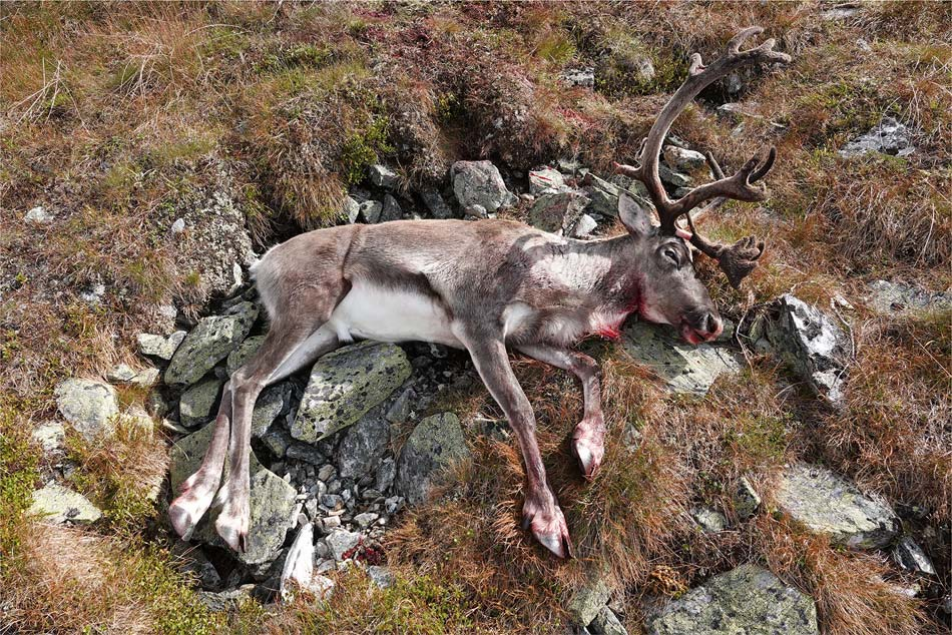
Sequenced specimen. Photograph of the wild alpine male specimen used for genome sequencing. The animal was legally shot during the ordinary hunting season on 29 August 2023 (Photo: Anne Eline Streitlien).

**Fig. 2:**
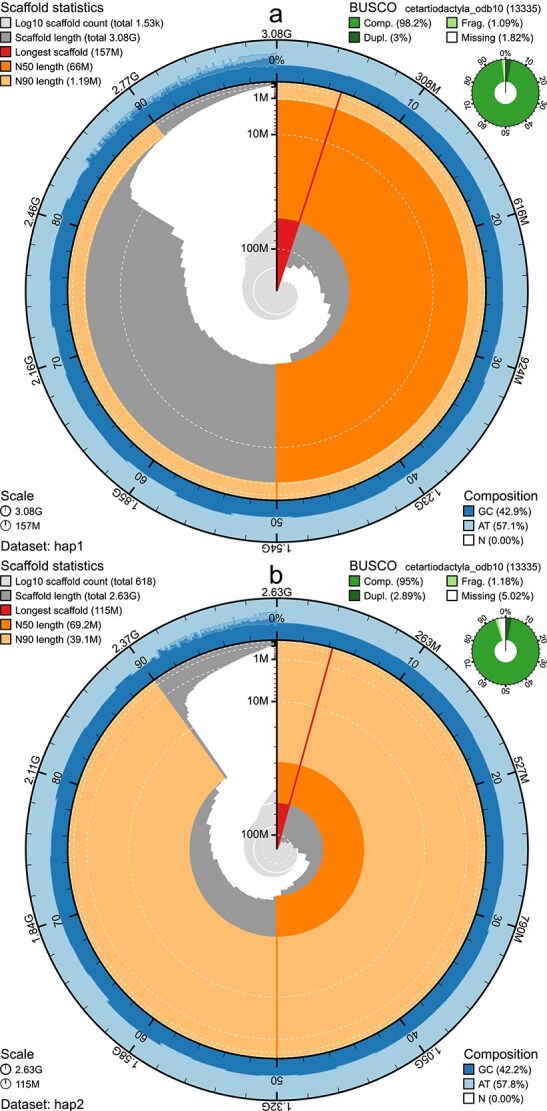
Metrics of the genome assemblies of *Rangifer tarandus tarandus,* (a) hap1 and (b) hap2. The BlobToolKit Snailplots show N50 metrics and BUSCO gene completeness. The two outermost bands of the circle signify GC versus AT composition at 0.1% intervals, with mean, maximum, and minimum. The third outermost shows the N90 scaffold length, while the fourth is N50 scaffold length. The line from middle to second outhermost band shows the size of the largest scaffold. All the scaffolds are arranged in a clockwise manner from largest to smallest, and shown in darker gray with white lines at different orders of magnitude, shown as a scale on the radius. The light gray shows the cumulative scaffold count. The scale inset in the lower left corner shows the total amount of sequence in the whole circle, and the fraction of the circle encompassed in the largest scaffold.

The genome from a wild alpine male reindeer (Fig. 1), had an estimated genome size of 2.42 Gbp, with 0.53% heterozygosity and a bimodal distribution based on the k-mer spectrum (Supplementary Fig. S1). The clear shoulder on the left signifies the k-mers corresponding to the heterozygous regions of the genome, whereas the main peak is from homozygous regions (Supplementary Fig. S1). A total of 28-fold coverage in PacBio single-molecule HiFi long reads and 85-fold coverage in Arima Hi-C reads resulted in two haplotype-separated assemblies. The final assemblies have total lengths of 3,081 and 2,633 Mb (Table 2 and Fig. 2), respectively. Both of these are slightly larger than the k-mer based estimation, a difference that might be due to k-mers occurring more than 10,000 times are being excluded. Pseudo-haplotypes one (hap1) and two (hap2) have scaffold N50 size of 66.0 and 69.2 Mb, respectively, and contig N50 of 30.8 and 40.7 Mb, respectively (Table 2 and Fig. 2). Thirty four autosomes were identified in both pseudo-haplotypes (numbered the same as in GCA_949782905.1; Svalbard reindeer) and the X and Y chromosomes were added to pseudo-haplotype one.

**Table 2:** Genome data for *Rangifer tarandus tarandus.*

Project accession data			
Species		*Rangifer tarandus tarandus*	
Specimen		mRanTar2	
NCBI taxonomy ID		86329	
BioProject		PRJEB86121	
BioSample ID		SAMEA117748988	
Isolate information		Male, heart tissue	
Raw data accessions			
PacBio HiFi reads		ERX14055576	1 PACBIO_SMRT (Revio) runs: 4.4 M reads, 83.9 Gb
Hi-C Illumina reads		ERX14055586	1 ILLUMINA (Illumina NovaSeq S4) run: 812 M pairs of reads, 254.4 Gb
Genome assembly metrics			
HiFi read coverage		28x	
Assembly accession		GCA_965653275	GCA_965653905
Assembly identifier		mRanTar2.1.hap1	mRanTar2.1.hap2
Span (Mb)		3,081	2,633
Number of contigs		1,766	845
Contig N50 length (Mb)		30.8	40.7
Longest contig (Mb)		90.9	84.0
Number of gaps		239	227
Number of scaffolds		1,527	618
Scaffold N50 length (Mb)		66.0	69.2
Longest scaffold (Mb)		156.6	115.2
Consensus quality (QV) compared with Hi-C (compared with HiFi)		47.3 (66.2)	47.9 (67.6)
Both assemblies		47.6 (66.8)	
k-mer completeness (percentage; compared with HiFi)		92.8 (93.2)	88.7 (88.4)
Both assemblies		98.2 (99.5)	
Percentage of assembly mapped to chromosomes		83.8	90.4
Comparisons (hap2 aligned to hap1)	Bases in alignment	2,449,903,016	
	Substitutions (percentage)	5,557,051 (0.23)	
	1 bp deletions	207,753	
	1 bp insertions	207,773	
	2 bp deletions	74,730	
	2 bp insertions	74,622	
	[3,50) deletions	138,479	
	[3,50) insertions	138,946	
	[50,1000) deletions	9,177	
	[50,1000) insertions	9,250	
	≥1,000 deletions	1,992	
	≥1,000 insertions	1,866	
Sex chromosomes (placed in hap1)		XY	
Organelles (placed in hap1)		MT	
Genome annotation metrics			
Number of protein-coding genes		37,998	36,977
Number of protein-coding genes with functional domain^a^		31,320	30,449
Number of protein-coding genes with gene names		19,898	19,520
Average length of CDS (bp)		1,065	1,072
Average number of exons per CDS		5.7	5.7
BUSCO^b^		C: 93.7% [S: 91.9%,D:1.7%], F: 0.7%, M: 5.6%, *n*: 13,335	C: 92.9% [S: 91.1%, D: 1.8%], F: 0.7%,M: 6.4%, *n*: 13,335

a Number of genes annotated with a functional domain as found by InterProScan

b BUSCO scores based on the cetartiodactyla BUSCO set using v5.8.3. C = complete, S = single copy, D = duplicated, F = fragmented, M = missing, *n* = number of orthologues in comparison.

In addition to the alpine reindeer, we reassembled the previously published Svalbard reindeer (Dussex et al. 2023) due to changes and improvements in the pipeline used, resulting in scaffold N50 size of 70.7 and 66.9 Mb, respectively, and contig N50 of 45.9 and 48.1 Mb, in pseudo-haplotypes one and two, respectively (Supplementary Table S1 and Table 3).

**Table 3:** Assembly metrics for various reindeer genome assemblies.

Assembly	Subspecies	Assembly length (Mb)	N50 contig (Mb)	N50 scaffold (Mb)	Chromosome level/sex chromosomes	Annotation available	Sequencing data used for contig creation
mRanTar2.1.hap1	*R. t. tarandus*	3,081	30.8	66.0	Yes/Yes	Yes	PacBio HiFi
mRanTar2.1.hap2	*R. t. tarandus*	2,633	40.7	69.2	Yes/No	Yes	PacBio HiFi
mRanTar1.2.hap1	*R. t. platyrhynchus*	2,990	45.9	70.7	Yes/Yes	Yes	PacBio HiFi
mRanTar1.2.hap2	*R. t. platyrhynchus*	2,821	48.1	66.9	Yes/No	Yes	PacBio HiFi
GCA_963422255.1	*R. t. fennicus (?)*	2,663	0.049	69.7	No/No		Illumina + Hi-C
GCA_902712895.1	*R. t. tarandus*	2,490	0.196	20.7	No/No		Illumina
GCA_022457185.1	Not specified	2,832	0.092	0.98	No/No		Illumina
GCA_019903745.2	*R. t. caribou*	2,577	0.164	54.4	Yes/No		Illumina, PacBio CLR and FISH
GCA_014898785.1	*R. t. granti*	2,537	0.231	9.35	No/No		Illumina
GCA_004026565.1	Not specified	2,897	0.078	0.089	No/No		Illumina

Most of the reindeer genome assemblies show good representation of complete BUSCO genes, with only one (GCA_004026565.1) having fewer than 90% complete genes (Table 4).

Hap1 had 98.0% and hap2 95.2% complete BUSCO genes using the mammalian lineage set (Fig. 2). When compared with a k-mer database of the Hi-C reads, hap1 had a k-mer completeness of 92.8%, pseudo-haplotype two of 88.7%, and combined they have a completeness of 98.2% (Supplementary Fig. S2, also see Supplementary Fig. S3). Further, hap1 has an assembly consensus quality value (QV) of 47.3 and pseudo-haplotype two of 47.9, where a QV of 40 corresponds to one error every 10,000 bp, or 99.99% accuracy compared with a k-mer database of the Hi-C reads (QV 66.2 and 67.6, respectively, compared with a k-mer database of the HiFi reads) (Table 2). The Hi-C contact map for the assemblies are shown in Supplementary Fig. S4 and show clear separation of the different chromosomes. GC-coverage plots for the assemblies are found in Supplementary Fig. S5, showing similar coverage in the chromosomes with some spread in GC content.

When comparing the two pseudo-haplotypes using minimap2, there are 5,557,051 SNPs differences (0.23% of the aligned sequence), 422,954 deletions in hap2 compared with hap1 ranging from 1 bp to more than 1,000 bp and 423,207 insertions from 1 bp to more than 1,000 bp in size (Table 2). A total of 37,998 and 36,977 protein-coding genes were annotated in pseudo-haplotype one and two, respectively (Table 2).

The assemblies generated in this study all have chromosome-level scaffolds, whereas only one other reindeer genome assembly has this (GCA_019903745.2) (Table 3). With the exception of the Svalbard reindeer assembly, none of the other publicly available genome assemblies have N50 contig length of more than 1 Mb. All the downloaded assemblies have shorter assembly lengths than the around 3,000 Mb for hap1 for both the alpine reindeer and Svalbard reindeer, in some cases more than 500 Mb shorter (Table 3).

## 4. Discussion

We have sequenced, assembled, and annotated chromosome-level, pseudo-haplotype separated genome assemblies from *R. tarandus tarandus* from the Rondane area in Norway. The Rondane population represents the native wild reindeer (Kvie et al. 2019). This is the second individual sequenced with long-read data, with the Svalbard reindeer done earlier (Dussex et al. 2023). For the Svalbard reindeer, we have reassembled the sequencing data previously generated and annotated the resulting pseudo-haplotype separated assembly, resulting in higher contig N50 lengths (45.9 and 48.1 Mb in the new version compared with 22.5 and 25.5 Mb in the original; Table 3), as well as better completeness with regards to BUSCO genes (98.1% and 95.2 compared with 96.3% and 94.1%, Table 3) (Dussex et al. 2023).

There are currently 8 genome assemblies for reindeer available in INSDC (i.e. ENA, NCBI, and DDBJ), including the two pseudo-haplotypes for Svalbard reindeer we published earlier (Dussex et al. 2023). Some of these are generated from individuals from specific sub-species, whereas this is not specified for others (Tables 3 and 4). Which genome assembly to use as a reference for a specific comparative or population genomic study will depend on the particulars of that study. For instance, even though the different assemblies vary substantially in contiguity (from 0.049 to 48.1 Mb N50 contig, almost 1,000-fold difference), the variation in complete BUSCO genes is much less (82.6% to 98.1%), illustrating that it is easier to assemble unique regions (e.g. genes) of a genome than the repetitive regions.

**Table 4:** BUSCO scores for various reindeer genome assemblies.

Assembly	Subspecies	Complete	Complete single	Complete duplicated	Fragmented	Missing
mRanTar2.1.hap1	*R. t. tarandus*	13,081 (98.1%)	12,681 (95.1%)	400 (3.0%)	157 (1.2%)	97 (0.7%)
mRanTar2.1.hap2	*R. t. tarandus*	12,653 (94.9%)	12,268 (92.0%)	385 (2.9%)	169 (1.3%)	513 (3.8%)
mRanTar1.2.hap1	*R. t. platyrhynchus*	13,086 (98.1%)	12,563 (94.2%)	523 (3.9%)	158 (1.2%)	91 (0.7%)
mRanTar1.2.hap2	*R. t. platyrhynchus*	12,689 (95.2%)	12,320 (92.4%)	369 (2.8%)	156 (1.2%)	490 (3.6%)
GCA_963422255.1	*R. t. fennicus* (?)	13,082 (98.1%)	12,731 (95.5%)	351 (2.6%)	177 (1.3%)	76 (0.6%)
GCA_902712895.1	*R. t. tarandus*	13,002 (97.5%)	12,657 (94.9%)	345 (2.6%)	190 (1.4%)	143 (1.1%)
GCA_022457185.1	Not specified	12,487 (93.7%)	11,998 (90.0%)	489 (3.7%)	502 (3.8%)	346 (2.5%)
GCA_019903745.2	*R. t. caribou*	12,841 (96.3%)	12,382 (92.9%)	459 (3.4%)	278 (2.1%)	216 (1.6%)
GCA_014898785.1	*R. t. granti*	12,943 (97.0%)	12,511 (93.8%)	432 (3.2%)	239 (1.8%)	153 (1.2%)
GCA_004026565.1	Not specified	11,010 (82.6%)	10,677 (80.1%)	333 (2.5%)	1,179 (8.8%)	1,146 (8.6%)

The previous Svalbard reindeer genome assembly (updated in this study) and this wild alpine reindeer genome are the only two of the available assemblies providing genome annotation at INSDC. Although there might be some annotations available for some of the other genomes, these are hard to locate since they are not linked from INSDC. The two genome assemblies presented here (wild alpine and Svalbard) are substantially more contiguous than other reindeer assemblies, have high amounts of complete BUSCO genes and available annotations, and will serve as excellent references for population genomic analyses. These annotated genomes will be valuable resources for studies addressing genes under selection in various scenarios such as between wild and domesticated reindeer and will facilitate further genomic research on ancestry, genetic diversity, inbreeding, genetic drift, population structure, domestication, feralization, and local adaptation of the reindeer, which may hopefully contribute towards preserving the last populations of wild alpine reindeer.

## Supplementary Material

reindeer_genome_resource_revision_supplement_esaf094

## Data Availability

Data generated for this study are available under ENA BioProject PRJEB86121. Raw PacBio sequencing data for the mountain reindeer (ENA BioSample: SAMEA117748988) are deposited in ENA under ERX14055576, whereas Illumina Hi-C sequencing data are deposited in ENA under ERX14055586. Pseudo-haplotype one can be found in ENA at PRJEB86097, whereas hap2 is PRJEB86119. The gene annotations are available at 10.5281/zenodo.15342678.
