# Supplementary material for: Chromosome-level genome assembly of Norwegian wild alpine reindeer (Rangifer tarandus tarandus)
Source: J Hered. 2025 Nov 3;117(3):557–65. doi: 10.1093/jhered/esaf094 (PMC13147171; doi:10.1093/jhered/esaf094)
Supplement: reindeer_genome_resource_revision_supplement_esaf094 [file reindeer_genome_resource_revision_supplement_esaf094.pdf]

Chromosome-level genome assembly of Norwegian wild alpine reindeer (*Rangifer tarandus tarandus*)

Authors: Ole K. Tørresen[1]*, Ave Tooming-Klunderud[1], Morten Skage[1], Anne Eline Streitlien[2], Olav Strand[3], Christer M. Rolandsen[3], Giada Ferrari[1], José Cerca[1,4,5], Atle Mysterud[1,3], Kjetill S. Jakobsen[1]*
*corresponding authors

Addresses:

[1] Centre for Ecological and Evolutionary Synthesis (CEES), Department of Biosciences, University of Oslo, P.O. Box 1066 Blindern, NO-0316 Oslo, Norway.

[2] Streitlievegen 131, 2580 Folldal.

[3] Norwegian Institute for Nature Research (NINA), P. O. Box 5685 Torgarden, NO-7485 Trondheim, Norway.

[4] Department of Bioinformatics and Genetics, Swedish Museum of Natural History, Box 50007, SE-104 05 Stockholm, Sweden.

[5] SciLifeLab, Karolinska Institutet Science Park, Box 1031, 17121 Solna, Sweden.

Supplementary Material

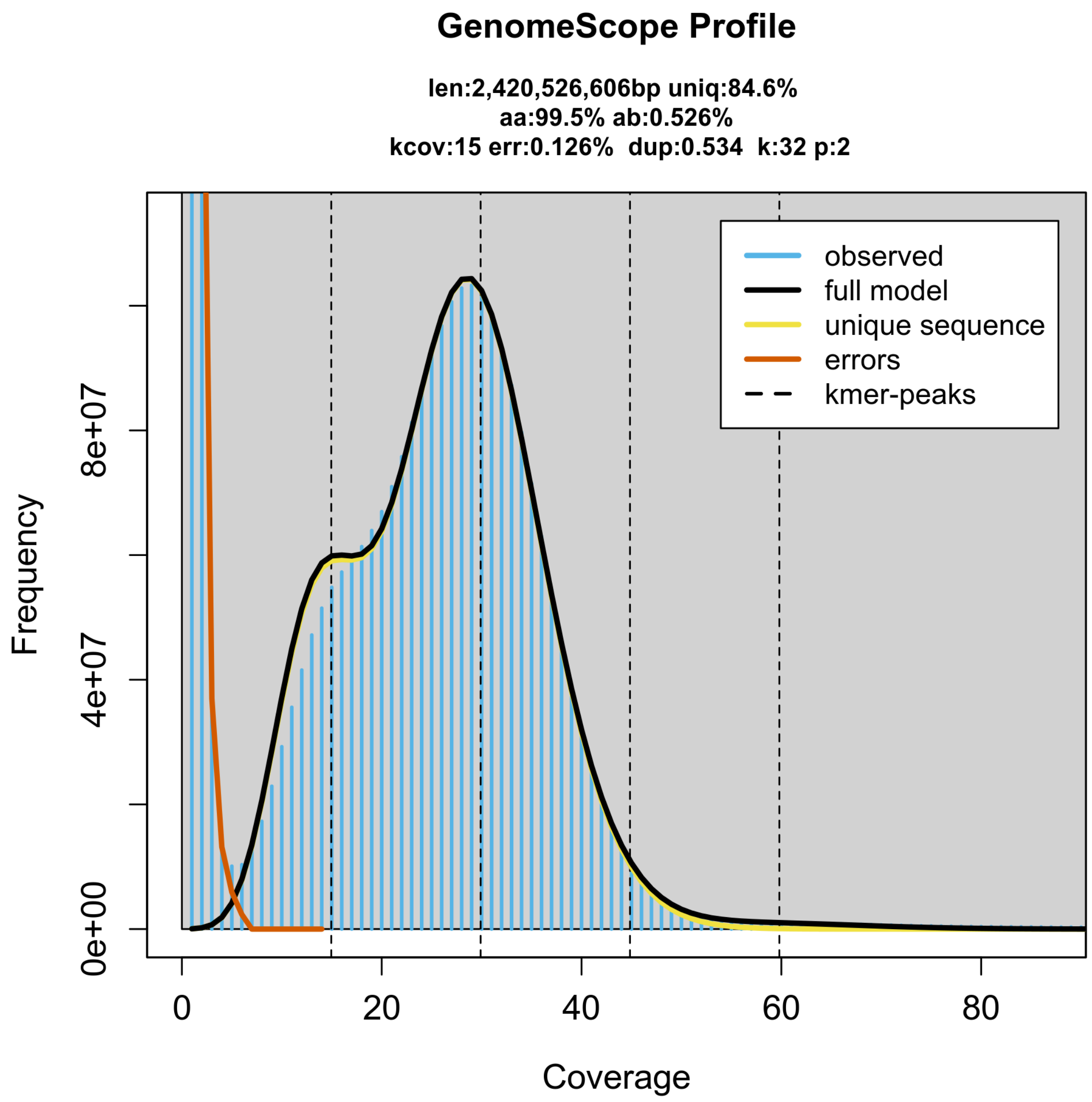

Supplementary Figure 1: GenomeScope profile of the HiFi reads from the sequenced individual. This analysis estimates a 2,420 Mb genome, with 0.53 % heterozygosity.

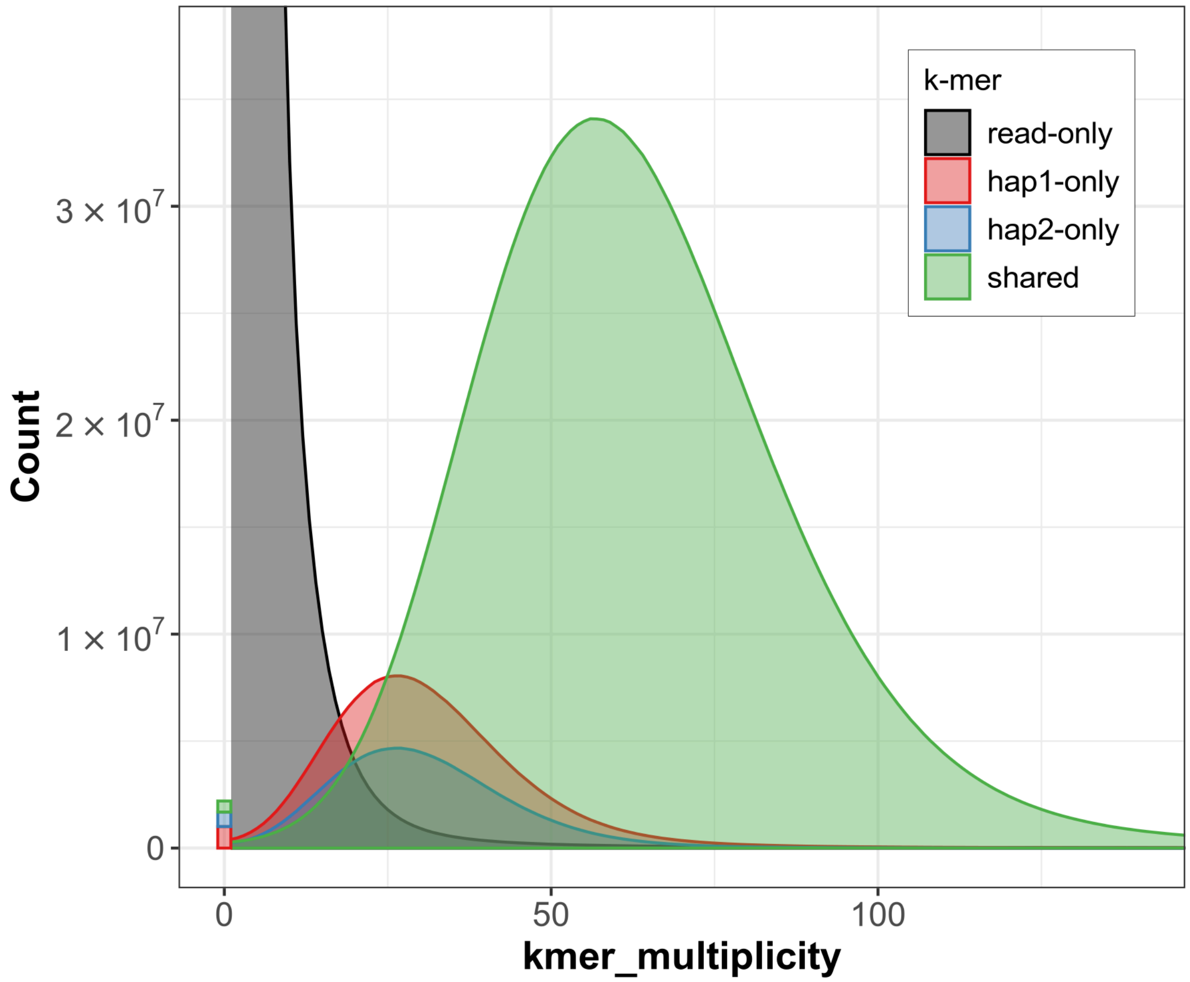

Supplementary Figure 2: K-mer copy-number spectrum analysis of *Rangifer tarandus tarandus* compared to k-mers from a database from the Hi-C reads. Assembly-specific k-mers are shown in red and blue, while k-mers shared by both pseudo-haplotypes are in green. The stack above 0 on the x-axis shows k-mers found in the assemblies, but not in the reads. Figure is generated by Merqury.

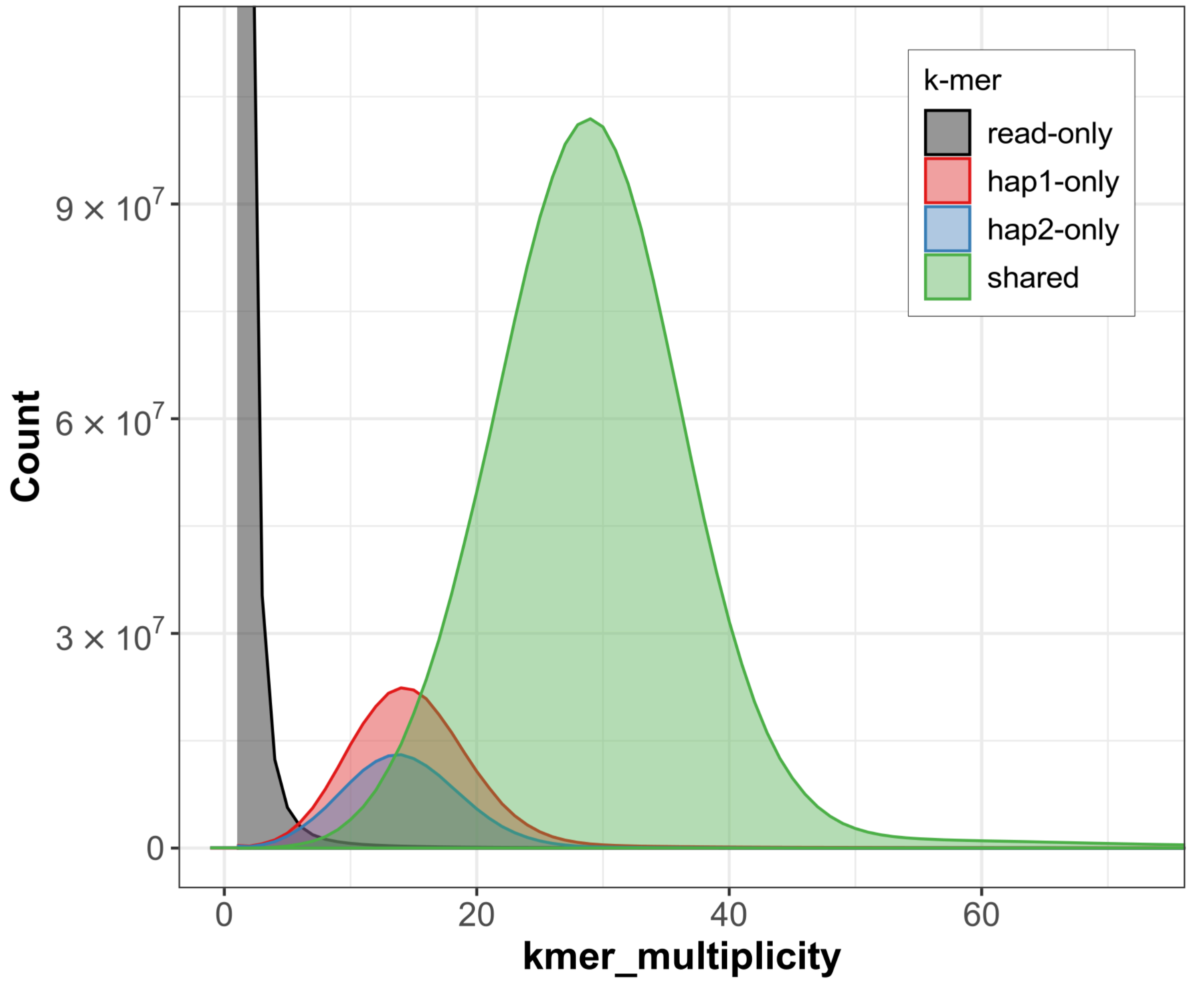

Supplementary Figure 3: K-mer copy-number spectrum analysis of *Rangifer tarandus tarandus* compared to k-mers from a database from the HiFi reads. Assembly-specific k-mers are shown in red and blue, while k-mers shared by both pseudo-haplotypes are in green. The stack above 0 on the x-axis shows k-mers found in the assemblies, but not in the reads. Figure is generated by Merqury.

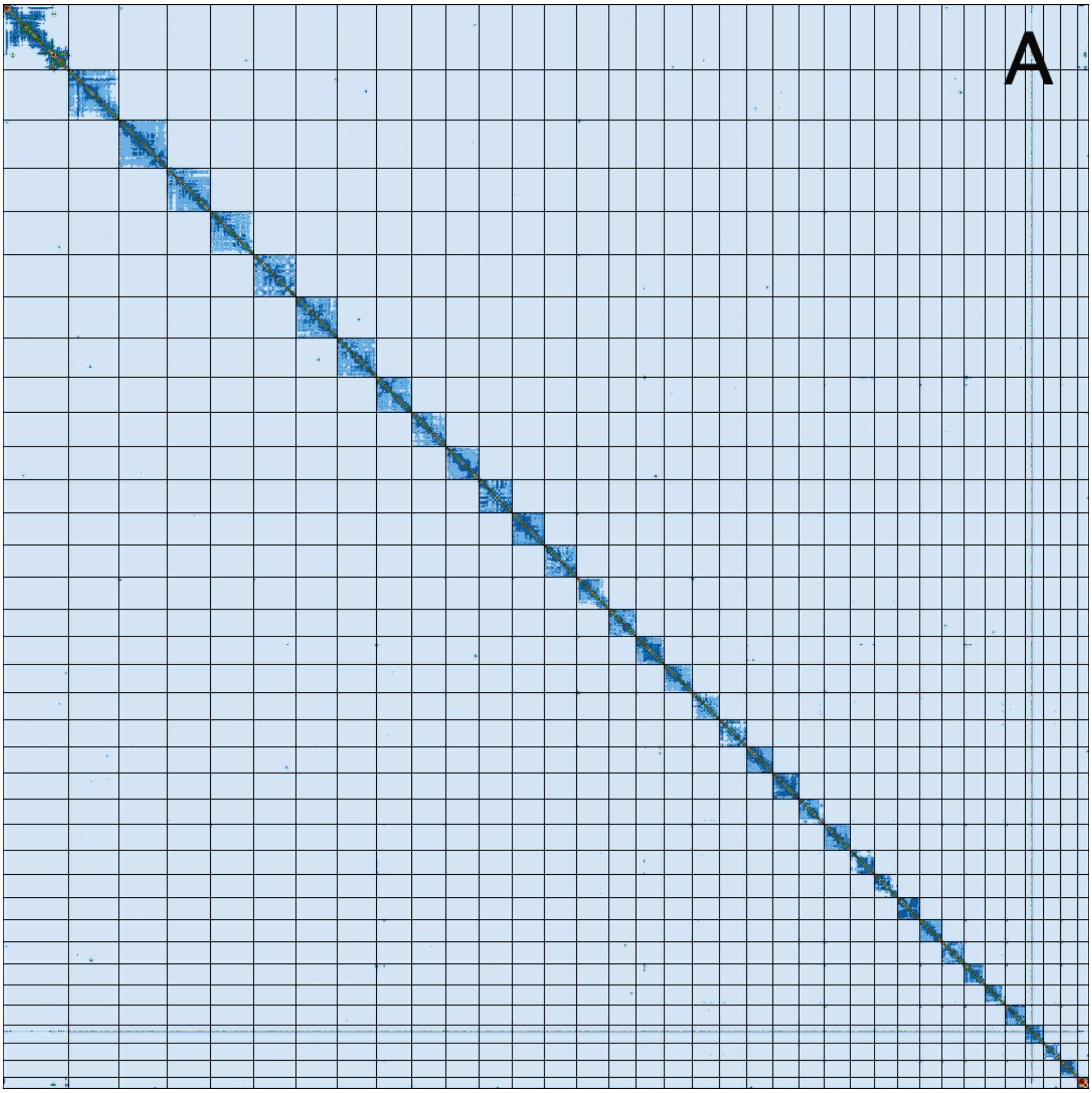
A

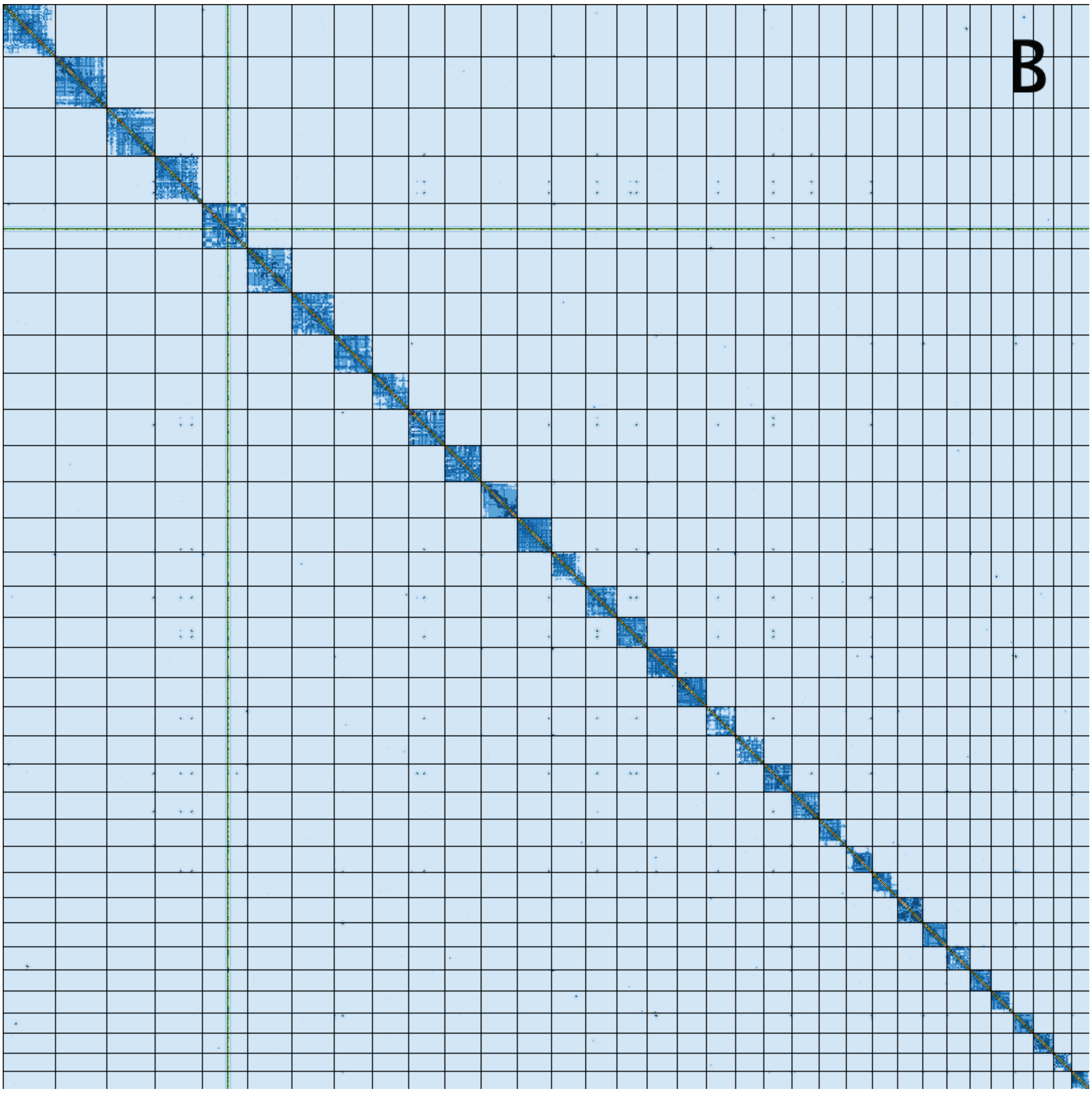

Supplementary Figure 4: Hi-C contact map of genome assemblies of *R. t. tarandus* for hap1 (A) and hap2 (B). The assemblies are visualized using PreTextSnapshot. Chromosomes are shown in order of size from left to right and top to bottom.

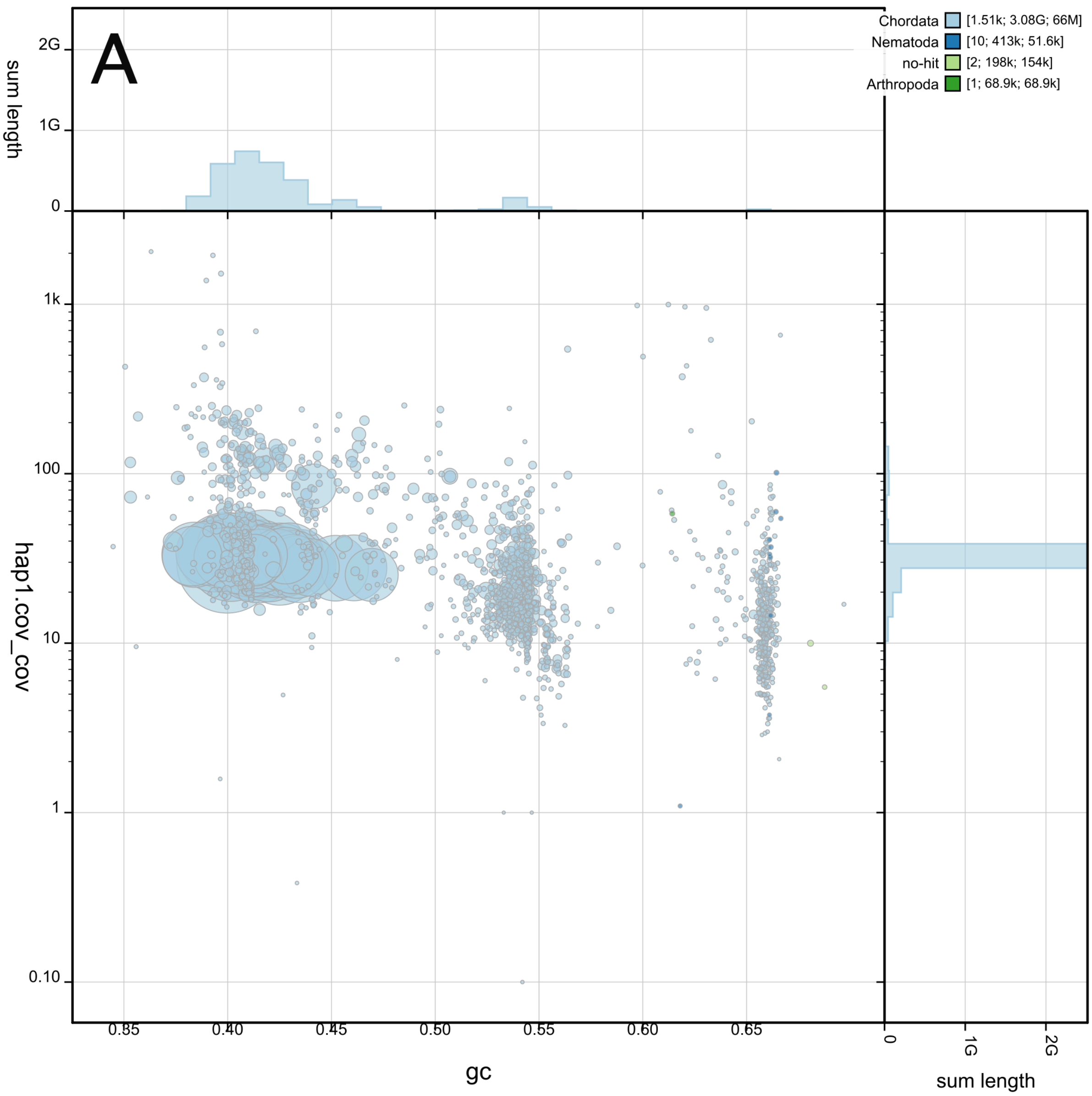
A
sum length
2G
1G
0
Chordata [1.51k; 3.08G; 66M]
Nematoda [10; 413k; 51.6k]
no-hit [2; 198k; 154k]
Arthropoda [1; 68.9k; 68.9k]
hap1.cov_cov
1k
100
10
1
0.10
0.35
0.40
0.45
0.50
0.55
0.60
0.65
gc
0
1G
2G
sum length

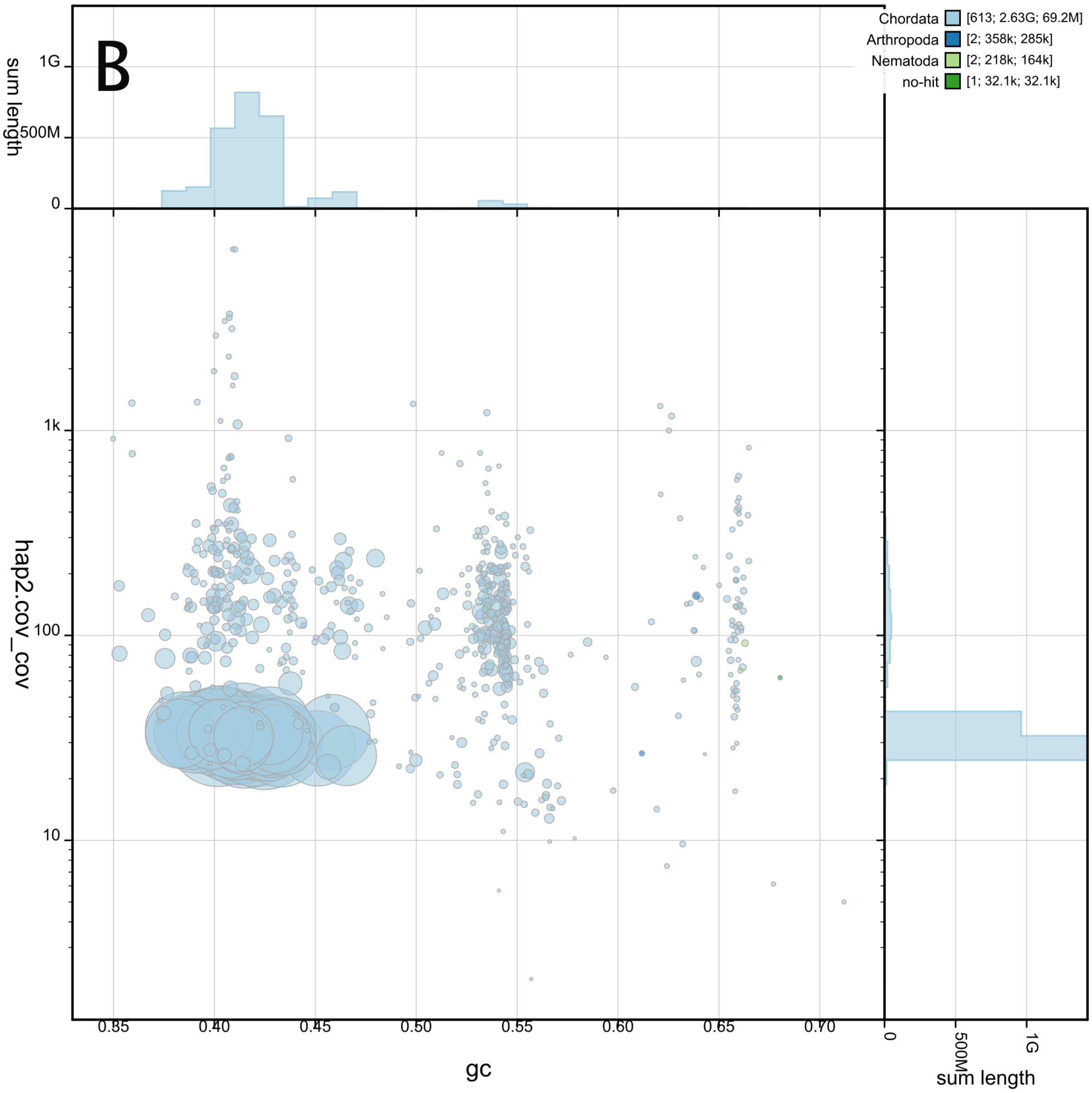

Supplementary Figure 5: BlobToolKit GC-coverage plots of genome assemblies of *Rangifer tarandus tarandus* hap1 (A) and hap2 (B). The scaffolds are coloured by phylum. The size of the circles are in proportion to the length of the scaffolds. Histograms show the distribution of scaffold length sum along each axis.

Supplementary Table 1: Genome data for *Rangifer tarandus platyrhynchus.*

**Project accession data**		
Species	*Rangifer tarandus platyrhynchus*	
Specimen	mRanTar1	
NCBI taxonomy ID	3082113	
BioProject	PRJEB65318	
Isolate information	Male, muscle	

**Raw data accessions**			
PacBio HiFi reads		3 PACBIO_SMRT (Sequel II) runs: 6.8 M reads, 98.1 Gb	
Hi-C Illumina reads		1 ILLUMINA (Illumina NovaSeq S4) run: 1113 M pairs of reads, 336.0 Gb	
**Genome assembly metrics**			
HiFi read coverage		35x	
Assembly identifier		mRanTar1.2.hap1	mRanTar1.2.hap2
Span (Mb)		2990	2821
Number of contigs		1576	1264
Contig N50 length (Mb)		45.9	48.1
Longest contig (Mb)		101.8	102.6
Number of gaps		145	51
Number of scaffolds		1431	1213
Scaffold N50 length (Mb)		70.7	66.9
Longest scaffold (Mb)		164.5	118.3
Consensus quality (QV) compared to Hi-C (compared to HiFi)		46.5 (64.4)	48.5 (66.8)
Both assemblies		47.4 (65.4)	
k-mer completeness (percentage; compared to HiFi)		96.1 (96.8)	92.0 (92.0)
Both assemblies		98.2 (99.3)	
Percentage of assembly mapped to chromosomes		88.2	84.7
Comparisons (hap2 aligned to hap1)	Bases in alignment	2,546,074,462	
	Substitutions (percentage)	2,425,311 (0.09)	
	1bp deletions	81,267	
	1bp insertions	82,423	
	2bp deletions	29,532	
	2bp insertions	29,027	
	[3,50) deletions	51,569	
	[3,50) insertions	51,343	
	[50,1000) deletions	5,168	

	[50,1000) insertions	5,116	
	>=1000 deletions	2,216	
	>=1000 insertions	1,939	
Sex chromosomes (placed in hap1)		XY	
Organelles (placed in hap1)		Not identified	
**Genome annotation metrics**			
Number of protein-coding genes		32,900	30,290
Number of protein-coding genes with functional domain**		26,960	24,930
Number of protein-coding genes with gene names		20,440	19,221
Average length of CDS (bp)		1,207	1,223
Average number of exons per CDS		6.6	6.8
BUSCO*		C:96.3%[S:93.6%,D:2.7%],F:0.7%,M:3.0%,n:13335	C:93.3%[S:91.5%,D:1.7%],F:0.7%,M:6.1%,n:13335

* BUSCO scores based on the cetartiodactyla BUSCO set using v5.8.3. C = complete [S = single copy, D = duplicated], F = fragmented, M = missing, n = number of orthologues in comparison.
**Number of genes annotated with a functional domain as found by InterProScan
